# Giant atypical lipomatous tumor/well-differentiated liposarcoma affects lower limb activity: A case report

**DOI:** 10.1097/MD.0000000000017619

**Published:** 2019-10-18

**Authors:** Haibo Zhan, Suixia Cao, Tian Gao, Bin Zhang, Xiaolong Yu, Lizhen Wang, Jin Zeng, Min Dai

**Affiliations:** a Department of Orthopedics, Multidisciplinary Therapy Center of Musculoskeletal Tumor, The First Affiliated Hospital of Nanchang University; b Medical School of Nanchang University; c Department of Pathology, The First Affiliated Hospital of Nanchang University, Nanchang, Jiangxi, P.R. China.

**Keywords:** atypical lipomatous tumor, diagnosis, liposarcoma, surgical therapy, well-differentiated/dedifferentiated

## Abstract

**Rationale:**

Liposarcomas are locally invasive mesenchymal soft tissue tumors; most deep liposarcomas are large. Liposarcomas have heterogeneous histomorphology, molecular and genetic characteristics, and clinical prognosis, making the diagnosis and treatment of giant liposarcomas difficult for bone tumor surgeons.

**Patient concerns:**

A 70-year-old man presented with a mass in the posterior part of his left lower extremity that was first noticed 3 years prior. The mass was initially fist sized but continued to grow and had been affecting lower limb mobility on presentation.

**Diagnoses:**

Computed tomography and magnetic resonance imaging revealed a large space-occupying lesion in the left thigh muscles, which was identified as a low-grade malignant tumor. Postoperative pathology results confirmed the diagnosis of atypical lipomatous tumor/well-differentiated liposarcoma (ALT/WDLPS).

**Interventions:**

The patient underwent open surgery to completely remove the tumor tissue and relieve pain.

**Outcomes:**

At the 10-month follow-up appointment, the patient had recovered well, function of the lower extremities had returned to normal, and no signs of recurrence or metastasis were noted.

**Lessons:**

Although ALT/WDLPS is a locally invasive tumor with good prognosis, delayed treatment is associated with increased tumor size, which can affect lower limb mobility. Therefore, we believe that extensive surgical resection of tumor tissue is a suitable treatment for all ALT/WDLPS cases in order to avoid possible local recurrence. In addition, for ALT/WDLPS tumors that are difficult to extensively excise, long-term follow-ups are necessary due to the possibility of recurrence.

## 1 Introduction

Soft tissue sarcoma is a heterogeneous disease comprising more than 50 histological subtypes with different biological behaviors. The most common subtype is liposarcoma, which is a malignant tumor of mesenchymal origin. Liposarcomas are histologically heterogeneous, and histological subtypes can predict patient outcomes. The recently updated World Health Organization classification of soft tissue and bone tumors identified the following 4 major liposarcoma subtypes: atypical lipomatous tumor/well-differentiated liposarcoma (ALT/WDLPS), dedifferentiated liposarcoma (DDLPS), myxoid liposarcoma, and pleomorphic liposarcoma.^[1]^ Dedifferentiated and pleomorphic liposarcomas are high-grade aggressive tumors with metastatic potential, whereas ALT/WDLPS and myxoid liposarcomas are low-grade tumors with a more indolent clinical course.^[2,3]^

ALT/WDLPS is a locally invasive, nonmetastatic cancer. ALT is synonymous with WDLPS, and the use of the term ALT is determined principally by tumor location and resectability.^[4]^ WDLPS occurring in a limb is often referred to as ALT because it does not metastasize and is easily cured. In contrast, WDLPS occurring in the retroperitoneum is more likely to recur or dedifferentiate; thus, the term WDLPS is used to emphasize its malignant features.^[5]^ ALT/WDLPS often occurs in middle-aged or elderly people. The most common site for ALT/WDLPS to occur is in deep soft tissues of the limbs, such as the thigh, retroperitoneum, mediastinum, and para-testicular tissues, and subcutaneous tissues.^[6]^ ALT/WDLPS is histologically characterized by atypical stromal cells and lipoblasts in mature fat, usually with prominent sclerotic components.^[7,8]^

In general, ALT/WDLPS is not a malignant transformation of a lipoma and there is no risk of metastasis unless dedifferentiation occurs. However, a delay in treating liposarcoma can result in an increase in the tumor size, which would ultimately affect the lower limb activity. Reports on giant ALT/WDLPS are relatively rare and reports describing complete patient data, as this report does, are even more rare. Here, we present a case of giant ALT/WDLPS in the deep left thigh of a patient.

## 2 Case presentation

On January 26, 2018, a 70-year-old Chinese man with a posterior mass in his left lower extremity presented to the outpatient clinic of our hospital. The patient could feel the borders of the mass 3 years prior when the mass was fist sized; however, the patient ignored it and did not undergo treatment. In the ensuing 3 years, the mass continued to increase in size and began to affect his lower limb activity to the point where the patient could not walk or work normally. The patient had no relevant history of injuries, past medical illnesses, or family history of major diseases.

**Figure 1:**
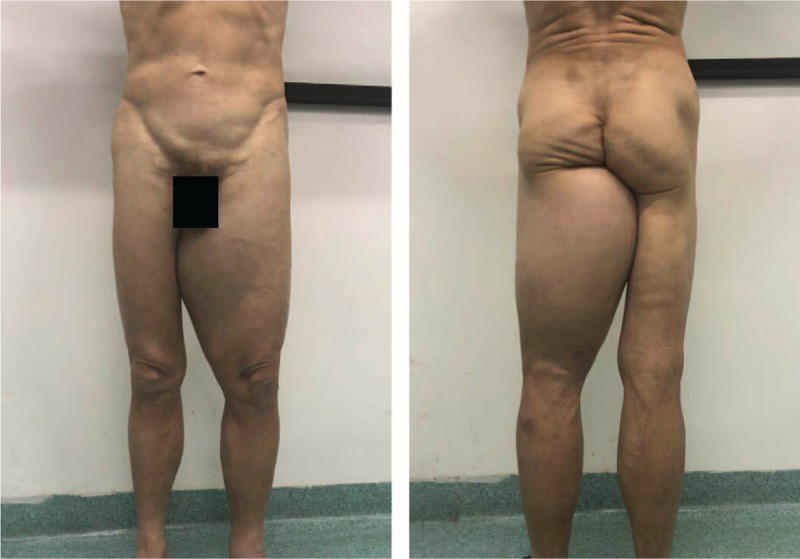
Giant tumor of the thigh. Enlargement of the tumor in the thigh affecting lower limb activity.

**Figure 2:**
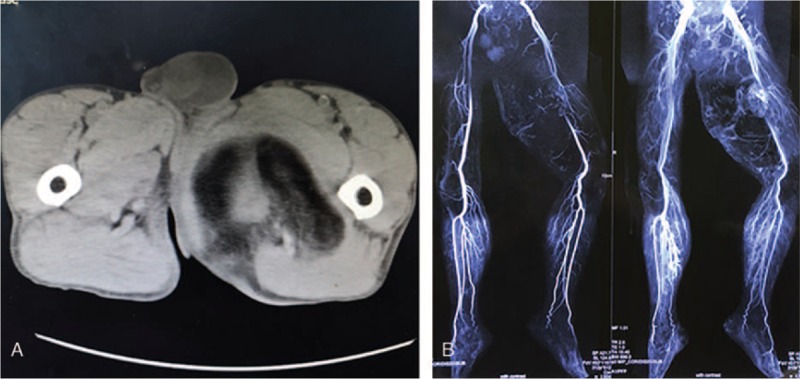
Preoperative CT and MRA findings. CT showed a very low-density shadow in the soft tissue inside the left thigh, showing a large amount of fat density (A). There were no significant abnormalities found on the MRA of the lower extremity vessels (B). CT = computed tomography, MRA = magnetic resonance angiography.

**Figure 3:**
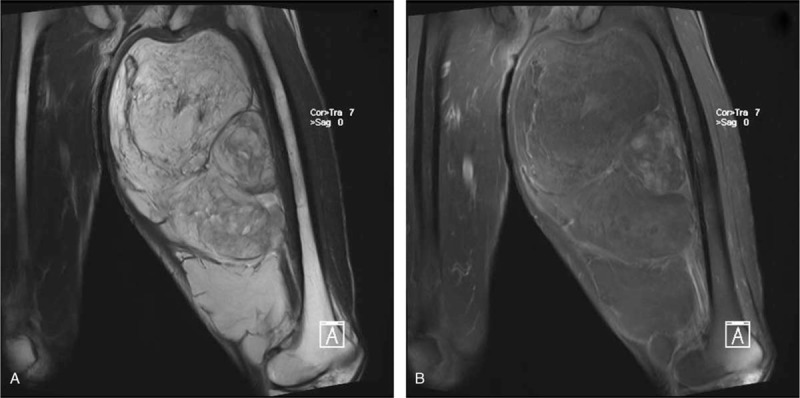
Preoperative magnetic resonance imaging findings. There was a huge agglomerate short T1 (A) and long T2 (B) signal in the left thigh muscle space, which was about 30 × 20 × 11 cm.

A physical examination revealed a large mass, measuring approximately 30 × 20 cm, on the posterior side of the left lower extremity (Fig. 1). The texture of the mass was tough and the boundary was unclear. No lower extremity edema was noted and the patient had normal dorsal artery pulsation. The carcinoembryonic antigen level (9.97 ng/mL) was higher than the normal range (0–7 ng/mL). The computed tomography scan revealed a very low-density shadow in the soft tissue inside the left thigh (Fig. 2A). No significant abnormalities were detected on magnetic resonance angiography imaging of the vessels of the lower extremities (Fig. 2B). However, magnetic resonance imaging revealed a huge agglomerate short T1 (Fig. 3A), long T2 (Fig. 3B) signal in the left thigh muscle space, which was approximately 30 × 20 × 11 cm in size. A pathological examination after core needle biopsy revealed that the tissue consisted of differentiated mature adipocytes with no atypical cells. Thus, we initially diagnosed the mass to be a low-grade malignant tumor, either lipoma or ALT/WDLPS.

**Figure 4:**
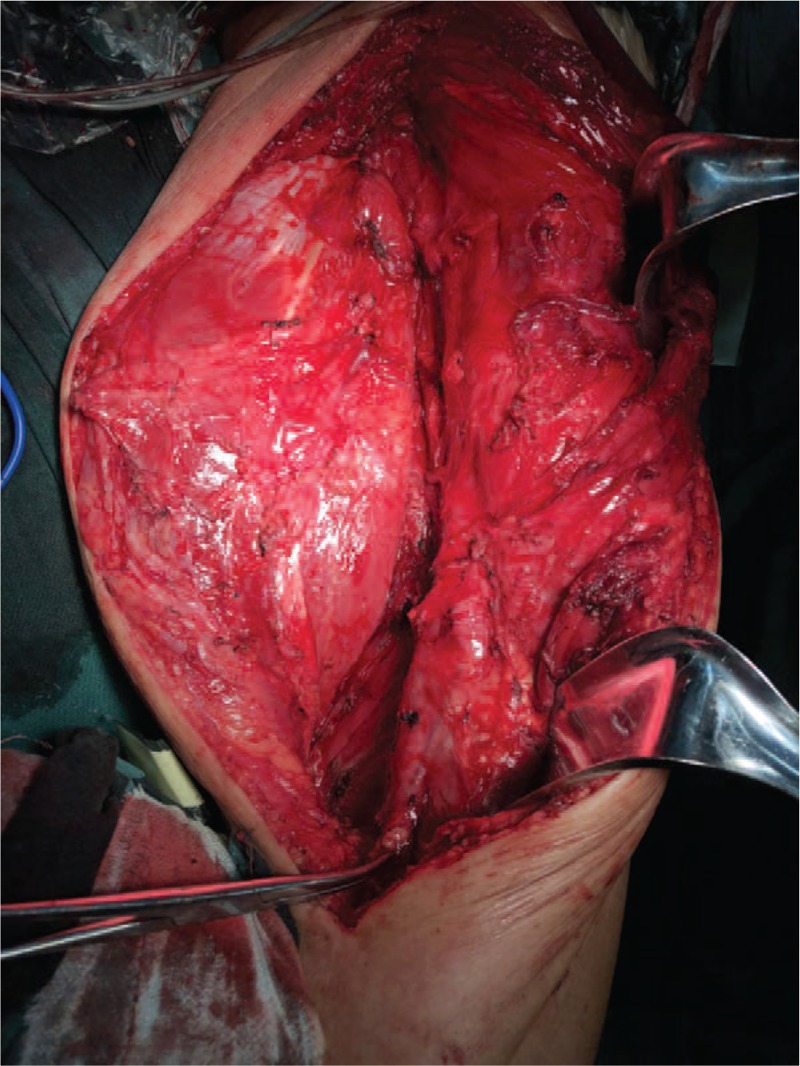
Intraoperative finding of the tumor. The soft tissue mass is located in the musculi tensor fascia latae, with a hard texture and clear borders.

**Figure 5:**
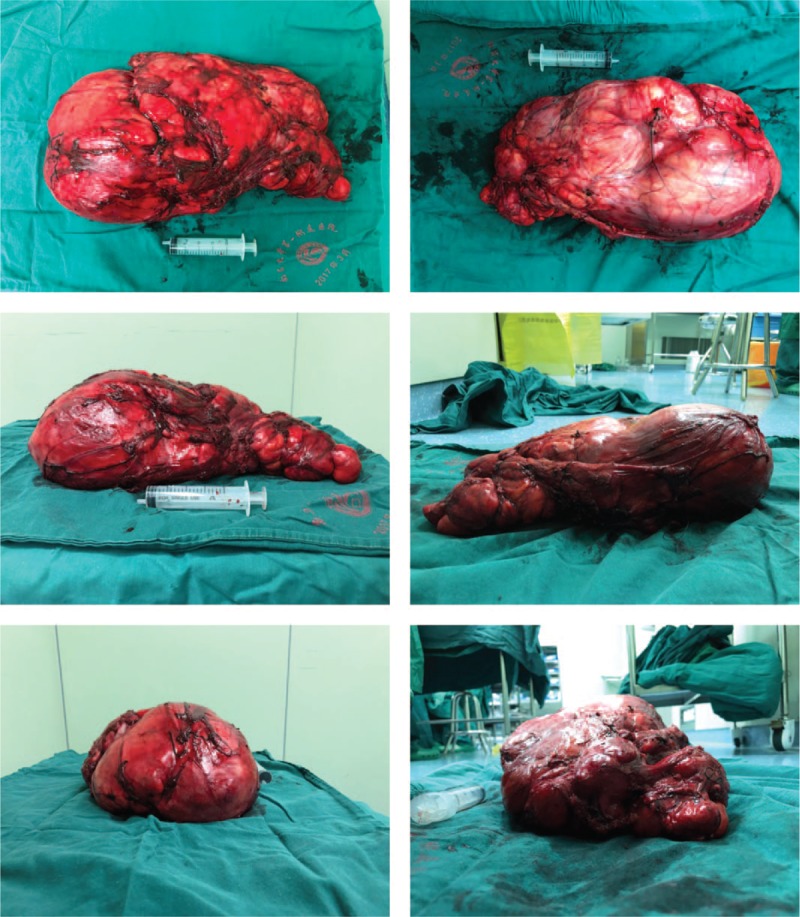
Macroscopic findings of the tumor. The size of the excised specimen is 37 × 23 × 11 cm.

**Figure 6:**
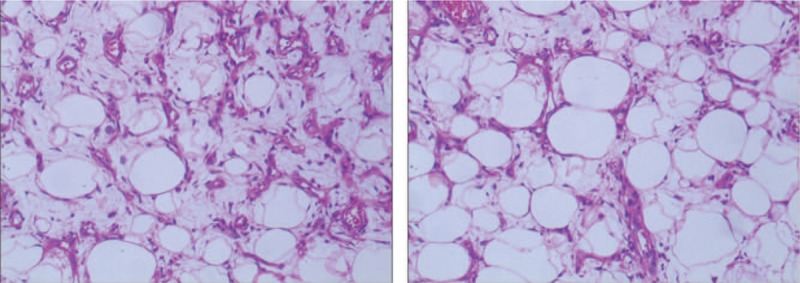
Microscopic findings of the tumor. The tumor is comprised adipose and some fibrous tissues. Mature fat cells in varying size, with scattered heterogeneous nuclear deep-stained mesenchymal cells and a small number of lipoblasts, were observed (×100).

To confirm the diagnosis and alleviate the patient's symptoms, the tumor was excised by an experienced bone tumor surgeon after all surgical contraindications were excluded. For the procedure, the patient was first administered general anesthesia and placed in the supine position. The surgeon took a medial approach to the left thigh. After cutting the skin, subcutaneous tissue, and superficial fascia, a soft tissue mass was observed in the fascia latae and the mass was observed to have a hard texture with clear borders (Fig. 4). The mass, which was fatty, lobulated, and had a complete envelope, was completely excised (Fig. 5). After rinsing the surgical area with distilled water, the area was filled with hemostatic cotton and a drainage tube was implanted. The muscles and tissues were layered and sutured. An external vacuum sealing drainage device was used. Finally, the patient's incision, wound, and thigh were wrapped with sterile dressing, thus completing the operation. After the operation, the patient's vital signs were stable and he returned to the ward. Postoperative pathological examination revealed that the tumor comprised adipose and some fibrous tissues. Mature fat cells of varying sizes with scattered heterogeneous nuclear deep-stained mesenchymal cells and a small number of lipoblasts were observed (Fig. 6). Immunohistochemical analysis results were positive for Ki-67, CD34, and S100, but they were negative for HMB45. Murine double minute 2 (MDM2) and cyclin-dependent kinase 4 (CDK4) expression was not analyzed. Oil red O staining revealed the presence of lipoblasts. Postoperative pathological examination confirmed the diagnosis of ALT/WDLPS. The patient was discharged 9 days after surgery. After discussions with oncologists, no adjuvant chemotherapy or radiation therapy was administered.

At the 10-month follow-up, the patient's incision had healed well, there was no pain or discomfort in the left thigh, and the lower extremity functions had returned to normal.

This study was approved by the ethical review committee of The First Affiliated Hospital of Nanchang University Medical School, and written informed consent was obtained from the patient for publication of this case report and accompanying images.

## 3 Discussion

Although accurate comparisons are not possible, the ALT/WDLPS tumor reported in this case is one of the larger ALT/WDLPS tumors described in the literature to date. Most deep, giant, fat-derived tumors are liposarcomas; however, in some cases, lipomas can reach comparable sizes.^[9]^ Lipomas are mature adipose tissues that do not contain lipoblasts or atypical stromal cells. Most lipomas are small in size and weigh only a few grams^[10]^; however, giant lipomas can be as large as 55 × 38 cm.^[11]^ Studies have reported intermittent claudication caused by giant lipomas due to compression of blood vessels.^[12]^ This complication was not observed in our case, mainly because the tumor occurred between the adductor and hamstring compartment and the main vessels are in the anterior compartment. Distinguishing between liposarcoma and lipoma solely based on imaging findings is difficult and the differential diagnosis relies on histopathological evaluation to assess mitotic activity, cell atypia, necrosis, and invasion.^[13,14]^ The most accurate diagnosis depends on immunohistochemical and molecular analyses.

The cytogenetic features of ALT/WDLPS are excess circular macrochromosomes containing amplified 12q13-15 sequences; amplification of MDM2 and CDK4 genes is the most constant in this region.^[15–17]^ The human MDM2 gene is located in the 12q13-14 segment of the chromosome, which regulates cell cycle progression in the transition from the G1/S phase and controls the differentiation and proliferation of normal cells.^[18]^ MDM2-encoded proteins can bind to the p53 protein and negatively regulate the p53 protein, thereby promoting tumorigenesis.^[19]^ CDK4 regulates the G1/M cell cycle transition. In vitro, inhibition of CDK4 proteins can lead to tumor proliferation arrest.^[20]^ The first step for diagnosing ALT/WDLPS is identifying histopathological features and combining that information with the corresponding clinical conditions. Immunohistochemical staining for MDM2 and CDK4 can be performed to identify ALT/WDLPS in the absence of complete histological images. Studies have shown that the highest level of sensitivity and specificity can be obtained by simultaneously using MDM2 and CDK4 immunohistochemistry to diagnose ALT/WDLPS.^[21]^ Another study reported that the sensitivity and specificity of the immunohistochemical marker MDM2 are lower than that of fluorescence in situ hybridization (FISH) detection of the MDM2 gene. Therefore, FISH can be used to identify ALT/WDLPS that is difficult to diagnose.^[22]^ In our case, postoperative pathological examination confirmed the tumor as an ALT/WDLPS; thus, the diagnosis was made without performing immunohistochemical analysis of MDM2. For some adipose-derived tumors that are difficult to diagnose, MDM2 and CDK4 can be identified by immunohistochemistry or FISH. It should be noted that there is genetic overlap between DDLPS and ALT/WDLPS. DDLPS is a biphasic tumor in which one component is WDLPS and the other component is nonfat-derived sarcoma with different histological levels. Both MDM2 and CDK4 can be amplified/overexpressed in both ALT/WDLPS and DDLPS; therefore, WDLPS and DDLPS cannot be distinguished based on the amplification and overexpression of MDM2 and CDK4.^[21]^ After the tumor is completely resected, histopathological features can be used to distinguish between ALT/WDLPS and DDLPS.

Preoperative biopsy is necessary for undefined solid tumors of the limb. Most patients with primary retroperitoneal WDLPS, which is preferably treated surgically, do not require core biopsy, because neoadjuvant radiotherapy or chemotherapy has never been shown to improve prognosis in these patients. The histology of sarcoma of the limbs is more diverse and neoadjuvant chemotherapy may be needed to treat such tumors; thus, biopsies are often performed before surgery.^[5]^ However, diagnosis can only be made after pathological evaluation of the surgically excised specimen because core needle biopsy results are prone to sampling errors. In our case, core needle puncture was routinely performed before surgery; however, the tumor was not diagnosed as ALT/WDLPS until it was completely excised.

ALT/WDLPS is mainly treated surgically because chemotherapy and radiotherapy are not sensitive. For nonmetastatic limb ALT/WDLPS, it is unclear whether local excision is sufficient. In 2011, the National Comprehensive Cancer Network Clinical Practice Guidelines recommended that for most soft tissue sarcomas, a margin of >1 cm should be maintained. However, another report suggested that because WDLPS in the limb is nonmetastatic and local control is >90%, surgeons can plan for an edge of <1 cm to optimize function and aesthetics.^[5]^ In our case, the large tumor made extensive resection difficult; hence, most of the area was excised along the tumor edge (Fig. 5).

There are some limitations to this study. Molecular testing for MDM2 and CDK4 expression via immunohistochemistry and/or FISH were not performed. Additionally, a 10-month follow-up period is not ideal. To affirm the absence of local recurrence, a follow-up period of 5 years would be necessary.

## 4 Conclusion

In conclusion, although there can be sampling errors, preoperative biopsies are necessary for fat-derived tumors of the limb. The first step in diagnosing ALT/WDLPS is identifying histopathological features after resecting intact tumors and combining that information with the corresponding clinical data. Therefore, we believe that extensive surgical resection of tumor tissue is a suitable treatment for all ALT/WDLPS to minimize the chance of local recurrence. In cases where extensive tumor excision is difficult, continuous follow-up is required because of the possibility that at least 10% of well-differentiated liposarcomas can dedifferentiate.

## Acknowledgments

The authors thank the patient and his family for allowing us to use the medical documentation and information that led to the present article. The authors also thank the assistance of the company Editage in Shanghai, which provided English language editing.

## Author contributions

**Data curation:** Suixia Cao.

**Formal analysis:** Tian Gao, Xiaolong Yu, Lizhen Wang.

**Funding acquisition:** Bin Zhang, Min Dai.

**Investigation:** Tian Gao, Xiaolong Yu, Lizhen Wang.

**Methodology:** Bin Zhang, Jin Zeng.

**Project administration:** Bin Zhang, Min Dai.

**Resources:** Jin Zeng, Min Dai.

**Writing – Original Draft:** Haibo Zhan, Suixia Cao, Tian Gao.

**Writing – Review & Editing:** Haibo Zhan, Jin Zeng, Min Dai.
